# Formation of todorokite from “*c*-disordered” H^+^-birnessites: the roles of average manganese oxidation state and interlayer cations

**DOI:** 10.1186/s12932-015-0023-3

**Published:** 2015-07-15

**Authors:** Huaiyan Zhao, Xinran Liang, Hui Yin, Fan Liu, Wenfeng Tan, Guohong Qiu, Xionghan Feng

**Affiliations:** Key Laboratory of Arable Land Conservation (Middle and Lower Reaches of Yangtse River), Ministry of Agriculture, College of Resources and Environment, Huazhong Agricultural University, Wuhan, 430070 China

**Keywords:** “*c*-disordered” H^+^-birnessite, Todorokite, Average manganese oxidation state, Interlayer cation

## Abstract

**Background:**

Todorokite, a 3 × 3 tectomanganate, is one of three main manganese oxide minerals in marine nodules and can be used as an active MnO_6_ octahedral molecular sieve. The formation of todorokite is closely associated with the poorly crystalline phyllomanganates in nature. However, the effect of the preparative parameters on the transformation of “*c*-disordered” H^+^-birnessites, analogue to natural phyllomanganates, into todorokite has not yet been explored.

**Results:**

Synthesis of “*c*-disordered” H^+^-birnessites with different average manganese oxidation states (AOS) was performed by controlling the MnO_4_^−^/Mn^2+^ ratio in low-concentrated NaOH or KOH media. Further transformation to todorokite, using “*c*-disordered” H^+^-birnessites pre-exchanged with Na^+^ or K^+^ or not before exchange with Mg^2+^, was conducted under reflux conditions to investigate the effects of Mn AOS and interlayer cations. The results show that all of these “*c*-disordered” H^+^-birnessites exhibit hexagonal layer symmetry and can be transformed into todorokite to different extents. “*c*-disordered” H^+^-birnessite without pre-exchange treatment contains lower levels of Na/K and is preferably transformed into ramsdellite with a smaller 1 × 2 tunnel structure rather than todorokite. Na^+^ pre-exchange, i.e. to form Na-H-birnessite, greatly enhances transformation into todorokite, whereas K^+^ pre-exchange, i.e. to form K-H-birnessite, inhibits the transformation. This is because the interlayer K^+^ of birnessite cannot be completely exchanged with Mg^2+^, which restrains the formation of tunnel “walls” with 1 nm in length. When the Mn AOS values of Na-H-birnessite increase from 3.58 to 3.74, the rate and extent of the transformation sharply decrease, indicating that a key process is Mn(III) species migration from layer into interlayer to form the tunnel structure during todorokite formation.

**Conclusions:**

Structural Mn(III), together with the content and type of interlayer metal ions, plays a crucial role in the transformation of “*c*-disordered” H^+^-birnessites with hexagonal symmetry into todorokite. This provides further explanation for the common occurrence of todorokite in the hydrothermal ocean environment, where is usually enriched in large metal ions such as Mg, Ca, Ni, Co and etc. These results have significant implications for exploring the origin and formation process of todorokite in various geochemical settings and promoting the practical application of todorokite in many fields.

**Graphical abstract:**
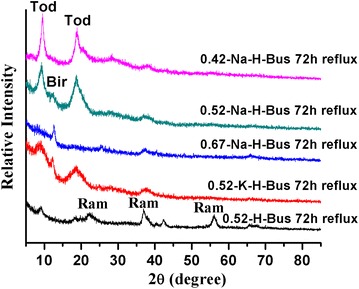
XRD patterns of Mg^2+^-exchanged and reflux treatment products for the synthetic “*c*-disordered” H^+^-birnessites.

## Background

Todorokite, a 3 × 3 tunnel structure with corner-sharing triple chains of MnO_6_ octahedra, is a naturally occurring manganese (Mn) oxide found in terrestrial Mn ore deposits, weathering products of manganese-bearing rocks, and marine Mn nodules [1–3]. Due to the superior characteristics of todorokite in ion exchange, specific surface area, thermal stability and molecule-sized tunnels [4], it has many potential industrial applications as molecular sieves, lithium-manganese-oxide cathode materials, heterogeneous catalysts and sensors [4–8], and it also plays an important role in cleaning up natural water and controlling the concentrations of heavy metals in soil solution [9].

Todorokites are often obtained by hydrothermal treatment or refluxing process from triclinic birnessite with a layered structure [4, 10, 11]. Oxidation of the Mn(II) located above or below vacant sites facilitates the formation of a stable todorokite-like structure during marine diagenesis and hydrothermal process [12]. Liu et al. [13] proposed that some octahedral Mn(III) from the layers of buserite could migrate into the interlayer and become corner-sharing octahedra that assist in the formation of the “wall” of the tunnels during hydrothermal heat treatment. Cui et al. [8, 14] also reported that the formation of todorokite under reflux conditions is closely related to the Mn(III) content and the content of Mn(III) in birnessite depends partly on the Mn AOS values. The degree of transformation of triclinic birnessite samples to todorokite decreases with the increasing of their Mn AOS values [8].

Recently Atkins et al. [15] found that “*c*-disordered” H^+^-birnessite with a poorly crystalline, hexagonal layer symmetry can also be transformed into todorokite under a mild reflux procedure. In addition, in the natural environment the microbial oxidation products of Mn(II) are mainly birnessite-like phases exhibiting poor crystallinity and hexagonal symmetry [16–19]. Thus, the formation of todorokite and poorly crystalline phyllomanganates are closely associated in natural marine environments. However, the effect of the preparative parameters on the transformation of “*c*-disordered” H^+^-birnessites into todorokite has not yet been explored.

Here we prepared “*c*-disordered” H^+^-birnessites with different Mn AOS by controlling the MnO_4_^−^/Mn^2+^ ratio in low-concentrated NaOH or KOH media. The transformation of “*c*-disordered” H^+^-birnessite to todorokite was achieved under a mild reflux condition, elucidating the roles of Mn AOS and the interlayer K^+^ and Na^+^ of the “*c*-disordered” H^+^-birnessite in the transformation. The results are expected to provide clues for understanding the transformation of poorly crystalline phyllomanganates with hexagonal symmetry to todorokite and shed light on the mechanisms of todorokite formation in earth-surface environment.

## Experimental methods

### Preparation of “*c*-disordered” H^+^-birnessites with different Mn AOS values

Synthesis of “*c*-disordered” H^+^-birnessite was performed following the method of Villalobos et al. [17]. A solution of 10 g of KMnO_4_ (0.1977 M) in 320 mL of distilled deionized water (DDW) was added slowly to a solution of 7.33 g of NaOH (0.51 M) solution in 360 mL of DDW while stirring. Subsequently, a solution of 23.26 g MnCl_2_ (0.3673 M) in 320 mL of DDW was added dropwise to the mixture while stirring vigorously to form precipitate. The suspension was left to settle for 4 h, following the supernatant was discarded. The pH value was 3.1. The remaining slurry was subsequently centrifuged at 10,000 rpm (Neofuge 23R) for 30 min and the resulting supernatant was discarded. The product was subjected to NaCl wash or Na^+^ pre-exchange following the procedures reported by Atkins et al. [15]. The centrifuged paste was mixed with 1 M NaCl, shaken for 1 h and centrifuged, the supernatant was discarded. This process was repeated 5 times. For the last 1 M NaCl wash the pH was adjusted to pH 8 via the drop-wise addition of 1 M NaOH and the suspension was shaken overnight. After centrifuging, the resulting paste was combined with DDW, shaken for 1 h and centrifuged. This DDW wash cycle was repeated 10 times. Following the final wash, the suspension was dialyzed for 3 days in 43 × 27 mm cellulose dialysis tubing. This sample is named 0.52-Na-H-Bir in this paper (0.52 was the ratio of Mn(VII)/Mn(II)).

The synthesis of 0.42-Na-H-Bir and 0.67-Na-H-Bir was similar to that of 0.52-Na-H-Bir, but the amounts added of MnCl_2_·4H_2_O were 30 g (0.4737 M) and 18.785 g (0.2966 M), respectively (the ratio of Mn(VII)/Mn(II) was 0.42 and 0.67). The synthesis of 0.52-H-Bir did not include the Na^+^ pre-exchange. The synthesis of 0.52-K-H-Bir was also similar to that of 0.52-Na-H-Bir, but used KOH solution as the material for synthesis and pH adjustment. The NaCl wash or Na^+^ pre-exchange was replaced by KCl wash or K^+^ pre-exchange.

### Transformation of “*c*-disordered” H^+^-birnessite to todorokite

Transformation of “*c*-disordered” H^+^-birnessite into todorokite was following a method adapted from Feng et al. [11, 20]. Approximately 20 g of wet “*c*-disordered” H^+^-birnessite slurry was dispersed in 1.5 L of 1 mol/L MgCl_2_ solution. After being fully exchanged for 24 h, the birnessites were converted into buserites (Bus). Birnessite and buserite are all the layer structure Mn oxides (phyllomanganates), and structurally related to each other. The former exhibits a 0.7 nm basal plane spacing due to the presence of single layer of water molecules and interlayer cations between the layers. When hydrated in the suspension or exchanged with larger hydrated cations (for instance, Mg^2+^, Ca^2+^), birnessite can be transformed into buserite with expanded basal plane spacing of 1.0 nm due to double layers of water molecules in the interlayers. In turn, upon drying or heating, buserite can be dehydrated and converted back into birnessite due to loss of one layer of water molecules. Then buserite solids were collected by centrifugation and re-suspended in 400 mL 1 mol/L MgCl_2_ solution and transferred to a 1 L triangular flask connected with a condensation device, then heated to reflux at 100°C and maintained at reflux conditions under magnetic stirring. Suspension aliquots were sampled at time intervals of 3, 6, 9, 12, 24, 48, 72 h, and 1 month. The produced minerals were washed until the conductance of the supernatant was below 20 μS/cm and freeze-dried.

### Characterization of “*c*-disordered” H^+^-birnessites and their products

XRD data were collected using Ni-filtered Cu Kα radiation (λ = 0.15418 nm) on a Bruker D8 Advance diffractometer equipped with a LynxEye detector. The diffractometer was operated at a tube voltage of 40 kV and a current of 40 mA with a scanning rate of 10°/min at a step size of 0.02°. Both buserite and todorokite have a basal d-spacing of ~1 nm. The former is not stable and can be transformed into the 0.7 nm phase (birnessite) during heating or dehydrating, whereas the latter has relatively high thermal stability [21]. To eliminate the interference of diffraction peaks of buserite on the identification of todorokite, the oriented samples spread on glass slices for the refluxed products were heated for 10 h at 140°C before XRD analysis [11].

The chemical composition of the samples was determined as follows: 0.1000 g of sample was dissolved in 25 mL of 0.25 mol/L NH_2_OH·HCl. The contents of Na and K were analyzed by a Varian Vista-MPX ICP-OES and the contents of Mn and Mg were measured using atomic absorption spectrophotometer (AAS). The average oxidation state (AOS) of Mn was determined using the oxalic acid-permanganate back-titration method.

Transmission electron microscopy (TEM) and high-resolution transmission electron microscopy (HRTEM) analyses of the crystal particles were conducted using a JEM-2100F electron microscope (JEOL, Japan) at an accelerating voltage of 200 kV. The samples were dispersed into absolute alcohol and ultrasonically vibrated prior to deposition on a holey carbon film, and then air-dried at room temperature.

The IR spectra of samples were recorded on a Bruker Vertex 70 FTIR spectrometer by making pellets with KBr. Samples were scanned 128 times between 4,000 and 400 cm^−1^ at a resolution of 4 cm^−1^.

## Results and analysis

### Characterization of synthesized “*c*-disordered” H^+^-birnessites

**Figure 1:**
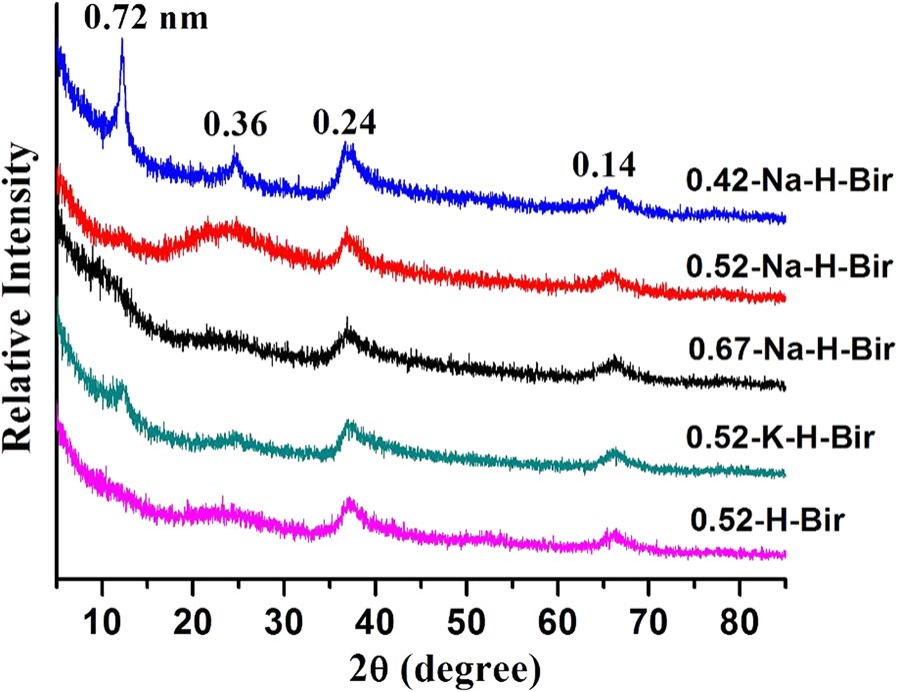
XRD patterns of the synthetic “*c*-disordered” H^+^-birnessites with different Mn(VII)/Mn(II) ratios pre-exchanged with Na^+^ or K^+^ or not.

The XRD patterns of the synthetic “*c*-disordered” H^+^-birnessites are shown on Figure 1. The 0.42-Na-H-Bir sample has primary characteristic peaks of birnessite at *d* spacings of 0.72, 0.36, 0.24, and 0.14 nm, which originate from the reflections of (001), (002), (100), and (110) planes (JPCDS 43-1456) [15], respectively. As the ratio of Mn(VII)/Mn(II) increases from 0.42 to 0.67, the 0.72 and 0.36 nm diffraction peaks gradually disappear, implying a decrease in the stacking period along the c* direction. The 0.52-K-H-Bir sample has a visible 0.72 nm diffraction peak, while this peak is absent in the 0.52-H-Bir sample. All these phases have two *hk0* reflections of the (100) and (110) planes at 0.24 nm and 1.4 nm. The *d*100/*d*110 peak intensity ratio (2.44/1.41 = 1.73) is close to $\sqrt 3$, which is in good agreement with that of “*c*-disordered” H^+^-birnessite synthesized by Villalobos et al. [17], indicating a hexagonal symmetry [22, 23]. This is further confirmed by the symmetrical 0.14 nm peak of these synthetic “*c*-disordered” H^+^-birnessites [23]. The 0.24 nm peak exhibits a degree of asymmetry on the high-angle side in all “*c*-disordered” H^+^-birnessites, which is typical of phyllomanganates that lack significant long-range ordering of the sheets [15, 24].

Chemical analyses and the average manganese oxidation states (AOS) of the synthetic “*c*-disordered” H^+^-birnessites are presented in Table 1. The Na^+^ content of the three types of Na-H-birnessite increases with the Mn(VII)/Mn(II) ratio increasing, and the content of K^+^ is far lower than that of Na^+^ in the birnessite samples pre-exchanged with Na^+^. The Mn contents of these Na-H-birnessites decrease and the Mn AOS values increase as the Mn(VII)/Mn(II) ratio increases. When the Mn(VII)/Mn(II) ratio is 0.42, 0.52 and 0.67, the Mn AOS values are 3.58, 3.63, and 3.74, respectively. The Na^+^ contents in 0.52-K-H-Bir and 0.52-H-Bir samples are very low, but the K^+^ content is considerably higher than the Na^+^ content in the 0.52-K-H-Bir sample and the K^+^ content in 0.52-H-Bir is one-quarter of that in the 0.52-K-H-Bir sample. The Mn contents and AOS values of these two samples are close to that of the 0.52-Na-H-Bir sample.

**Table 1:** Chemical analyses and average manganese oxidation states of the synthetic “*c*-disordered” H^+^-birnessites

Reaction condition	Ratio of MnO_4_^−^/Mn^2+^	Sample	Na (mmol/g)	K (mmol/g)	Mn (mmol/g)	AOS
KMnO_4_ + NaOH + MnCl_2_ (NaCl pre-exchange)	0.42	0.42-Na-H-Bir	1.61	0.20	9.84	3.58
KMnO_4_ + NaOH + MnCl_2_ (NaCl pre-exchange)	0.52	0.52-Na-H-Bir	1.64	0.03	9.68	3.63
KMnO_4_ + NaOH + MnCl_2_ (NaCl pre-exchange)	0.67	0.67-Na-H-Bir	2.29	0.05	8.78	3.74
KMnO_4_ + NaOH + MnCl_2_ (KCl pre-exchange)	0.52	0.52-K-H-Bir	0.01	2.43	9.15	3.66
KMnO_4_ + NaOH + MnCl_2_	0.52	0.52-H-Bir	0.07	0.61	9.56	3.64

**Figure 2:**
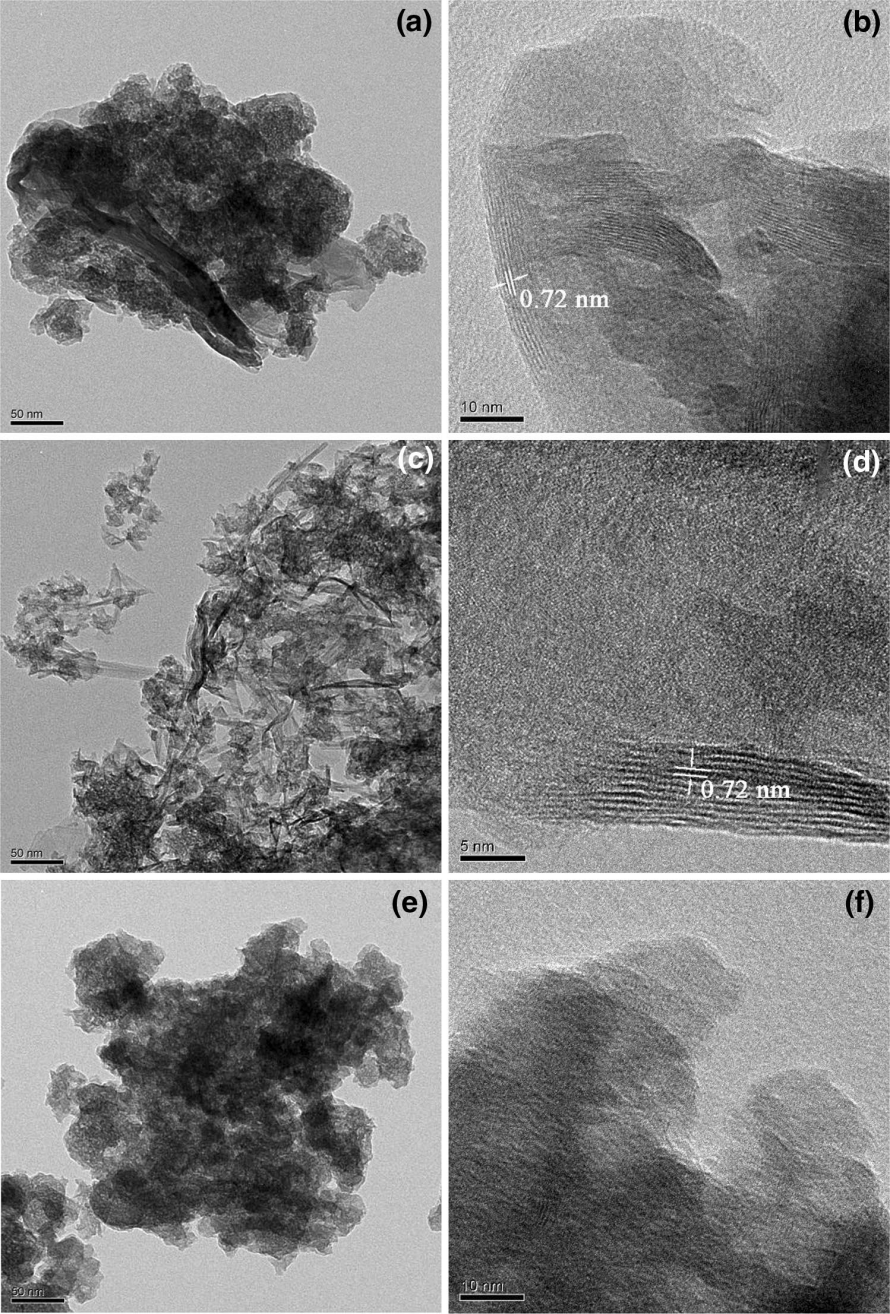
HR-TEM images of “*c*-disordered” Na-H-birnessites with different Mn(VII)/Mn(II) ratios. **a**, **b** 0.42-Na-H-Bir; **c**, **d** 0.52-Na-H-Bir; **e**, **f** 0.67-Na-H-Bir.

HR-TEM images of “*c*-disordered” Na-H-birnessites with different Mn(VII)/Mn(II) ratios are presented in Figure 2. The 0.42-Na-H-Bir sample contains aggregations of platy crystals with large size and multiple stacked layers (Figure 2a, b). The fringes of layer stacking are arranged along the *c*-axis direction (001), up to more than 10 nm stacking thickness with ~0.72 nm interlayer spacing. In addition, bending or curling of the layers, as reported in δ-MnO_2_ [25, 26], can also be clearly seen. When the Mn(VII)/Mn(II) ratio of Na-H-birnessite increases to 0.52, the birnessite consists of aggregations of small, thin plate-like crystals (Figure 2c, d), with a layer stacking thickness of ~5 nm. When the Mn(VII)/Mn(II) ratio of Na-H-birnessite increases to 0.67, the material formed even smaller and thinner platy crystals that are tightly aggregated together, and stacked layers of less than 5 nm are hardly observed (Figure 2e, f). Therefore, it is suggested that the crystallite sizes of the “*c*-disordered” Na-H-birnessites decrease with increasing Mn AOS, or with an increasing ratio of Mn(VII)/Mn(II) during synthesis.

**Figure 3:**
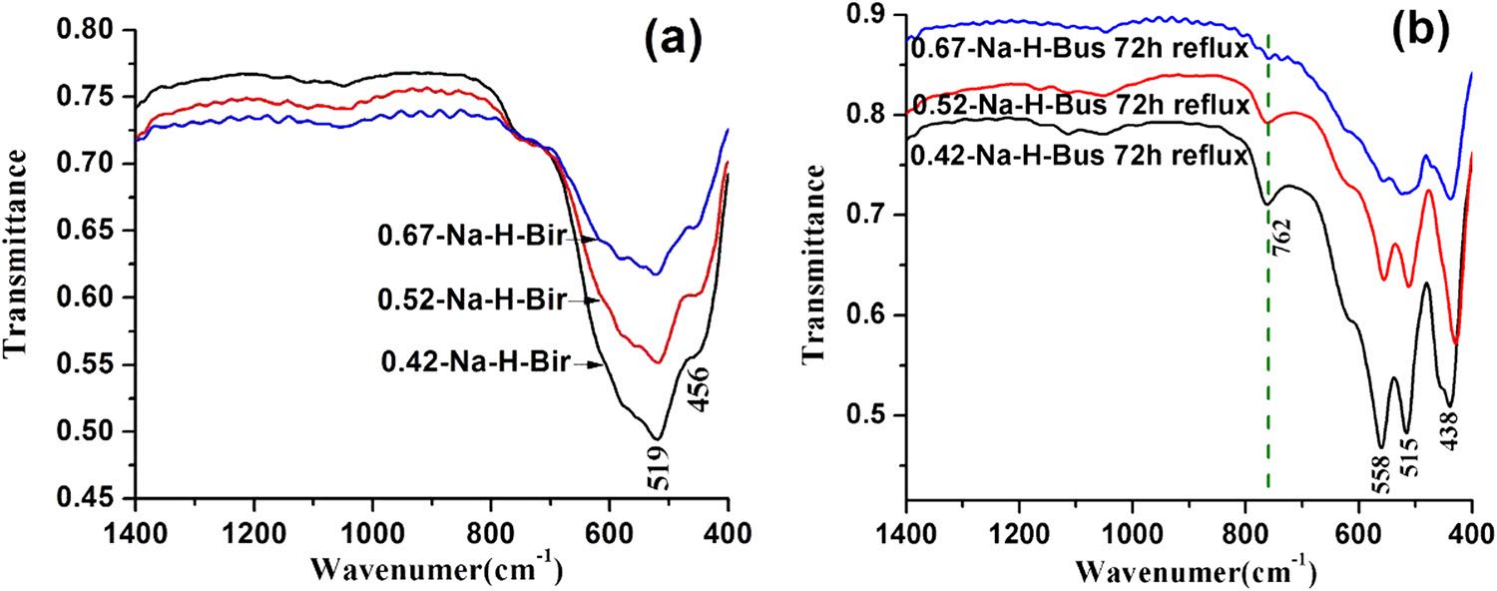
FTIR spectra of “*c*-disordered” Na-H-birnessites with different Mn(VII)/Mn(II) ratios (**a**) and their products after Mg-exchange and reflux treatment for 72 h (**b**).

The IR spectra of all synthetic “*c*-disordered” Na-H-birnessite samples in the range from 1,400 to 400 cm^−1^ are shown in Figure 3. The IR absorption bands around 500 and 450 cm^−1^ are diagnostic for the birnessite structure [10, 27–30]. The “*c*-disordered” Na-H-birnessites have two main IR bands at around 519 and 456 cm^−1^ (Figure 3a), which are in good agreement with the characteristic IR bands of birnessites. For different Mn(VII)/Mn(II) ratios of “*c*-disordered” Na-H-birnessites, the position of the characteristic adsorbed bands scarcely change, while the intensity becomes weaker as the Mn AOS value increases. Because the IR bands between 400 and 800 cm^−1^ can be assigned to Mn–O lattice vibration, changes in the position and intensity of these characteristic adsorbed bands can be attributed to changes in the sub-structure of the MnO_6_ octahedral layer [8]. In addition, the intensity of the adsorbed bands may be related to the crystallinity of the synthetic “*c*-disordered” Na-H-birnessites. With decreases in Mn AOS value of “*c*-disordered” Na-H-birnessites, the IR bands become stronger (Figure 3), indicating increasing crystallinity. This is consistent with the XRD and TEM results.

### Transformation from “*c*-disordered” H^+^-birnessite to buserite and todorokite

**Figure 4:**
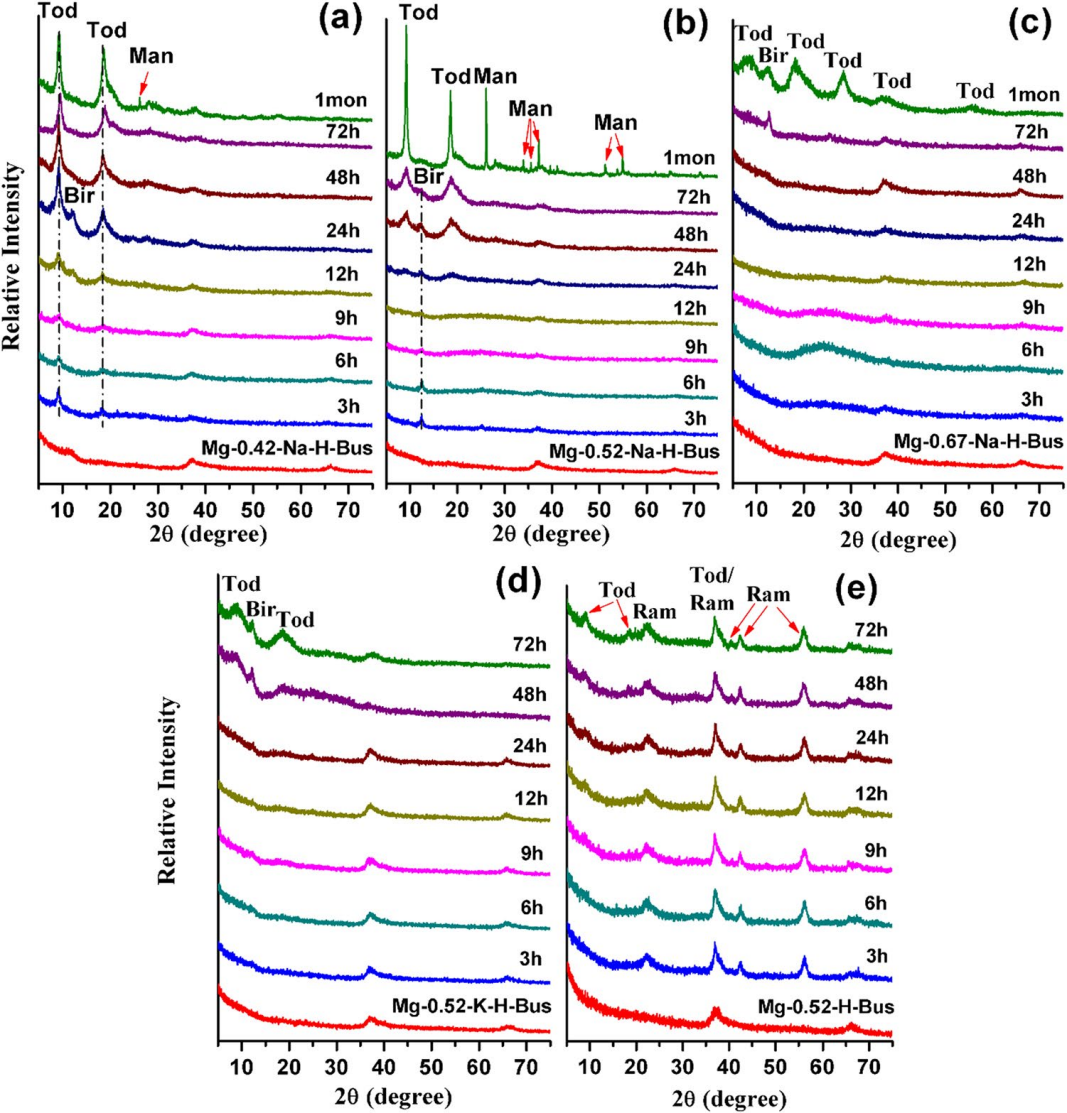
XRD patterns of Mg^2+^-exchanged intermediates and reflux products for the synthetic “*c*-disordered” H^+^-birnessites at different times. **a** 0.42-Na-H-Bir, **b** 0.52-Na-H-Bir, **c** 0.67-Na-H-Bir, **d** 0.52-K-H-Bir, **e** 0.52-H-Bir. *Tod* todorokite, *Bir* birnessite, *Man* manganite, *Ram* ramsdellite.

Golden et al. [10] reported that certain divalent cations, such as Mg^2+^, Ca^2+^, and Ni^2+^, possess larger hydrated ion radii and higher hydration energies compared to alkaline metal ions (Na^+^, K^+^, etc.). Therefore these divalent cations, through ion exchange, can readily expand the 0.7 nm basal plane spacing of birnessite to 1.0 nm of buserite which can be stabilized in the high humidity. After Mg^2+^-exchanged for 24 h, the birnessites were converted into buserites. The XRD patterns of Mg^2+^-exchanged “*c*-disordered” H^+^-birnessites are similar to those of their precursors (Figure 4). All XRD patterns of buserites have 0.24 and 0.14 nm diffraction peaks, and 0.42-Na-H-Bus (Mg^2+^-exchanged 0.42-Na-H-Bir) has an additional weak 0.72 nm reflection. The elemental composition indicates that nearly all the interlayer Na^+^ in “*c*-disordered” H^+^-birnessites is completely replaced by Mg^2+^, but in the 0.52-K-H-Bus and 0.52-H-Bus samples, part of the interlayer K^+^, 12.6 and 18.9%, respectively, is not replaced by Mg^2+^ (Table 2). The Mg^2+^ contents of these Na-H-buserites increase and the Mn contents decrease with increasing Mn(VII)/Mn(II) ratio during synthesis (Table 2).

**Table 2:** Elemental compositions of the synthetic “*c*-disordered” H^+^-birnessites after exchanged with Mg^2+^ for 24 h

Sample	Na (mmol/g)	K (mmol/g)	Mg (mmol/g)	Mn (mmol/g)	K/Mn	Mg/Mn	C/Mn*
0.42-Na-H-Bus	0.01	0.05	1.86	9.55	0.005	0.195	0.396
0.52-Na-H-Bus	0.02	0.04	2.34	8.56	0.005	0.273	0.554
0.67-Na-H-Bus	0.01	0.04	2.43	7.64	0.005	0.318	0.643
0.52-K-H-Bus	0	0.26	1.82	7.78	0.033	0.234	0.501
0.52-H-Bus	0.02	0.11	1.00	9.13	0.012	0.110	0.233

* Equivalent charge of metal ions per Mn.

Over the course of the reflux treatment of 0.42-Na-H-Bus, the characteristic diffraction peaks of todorokite appear at 3 h in the XRD pattern (Figure 4a). With reflux time prolonged to 48 h, the peaks of birnessite disappear leaving only strong todorokite XRD reflections, indicating that the 0.42-Na-H-Bus has been completely converted to todorokite. With a prolonged reflux time of 1 month, a very weak diffraction peak of manganite at 0.34 nm appears besides slightly strengthened those of todorokite (Figure 4a). For the 0.52-Na-H-Bus sample under reflux treatment, broad todorokite peaks and a very weak birnessite peak are present in the XRD patterns at reflux times of 48–72 h. As the reflux time is extended to 1 month, the birnessite peaks disappear and the characteristic peaks of todorokite become much stronger, while peaks of low-valence manganite (γ-MnOOH, JPCDS 8–99) are observed (Figure 4b). When the Mn(VII)/Mn(II) ratio increases to 0.67, only the characteristic peaks of birnessite are observed at a reflux time of 72 h, indicating that todorokite is not formed. At a reflux time of 1 month, the weak characteristic peaks of todorokite are visible with similar intensity to those of birnessite, suggesting that part of the 0.67-Na-H-Bus has been converted to todorokite (Figure 4c).

Furthermore, it is also observed that 0.52-K-H-Bus and 0.52-H-Bus after reflux treatment are partially transformed into todorokite but to a lesser extent compared to 0.52-Na-H-Bus, the corresponding Na^+^ pre-exchanged precursor. The intensities of the characteristic peaks of todorokite and birnessite in the XRD pattern of 0.52-K-H-Bus after reflux for 72 h (Figure 4d) are close to those of 0.52-Na-H-Bus refluxed for 48 h (Figure 4b). This suggests that 0.52-K-H-Bus is less favorable for the transformation than 0.52-Na-H-Bus. When 0.52-H-Bus (without Na^+^ or K^+^ pre-exchange treatment) is refluxed for 3 h, the characteristic peaks of ramsdellite (1 × 2 tunnel-structured γ-MnO_2_, JPCDS 44-0142) appear, while very weak XRD reflections of todorokite can be discerned after 12 h. With increasing reflux time, the intensities of the peaks of todorokite and those of ramsdellite remain nearly unchanged (Figure 4e).

**Figure 5:**
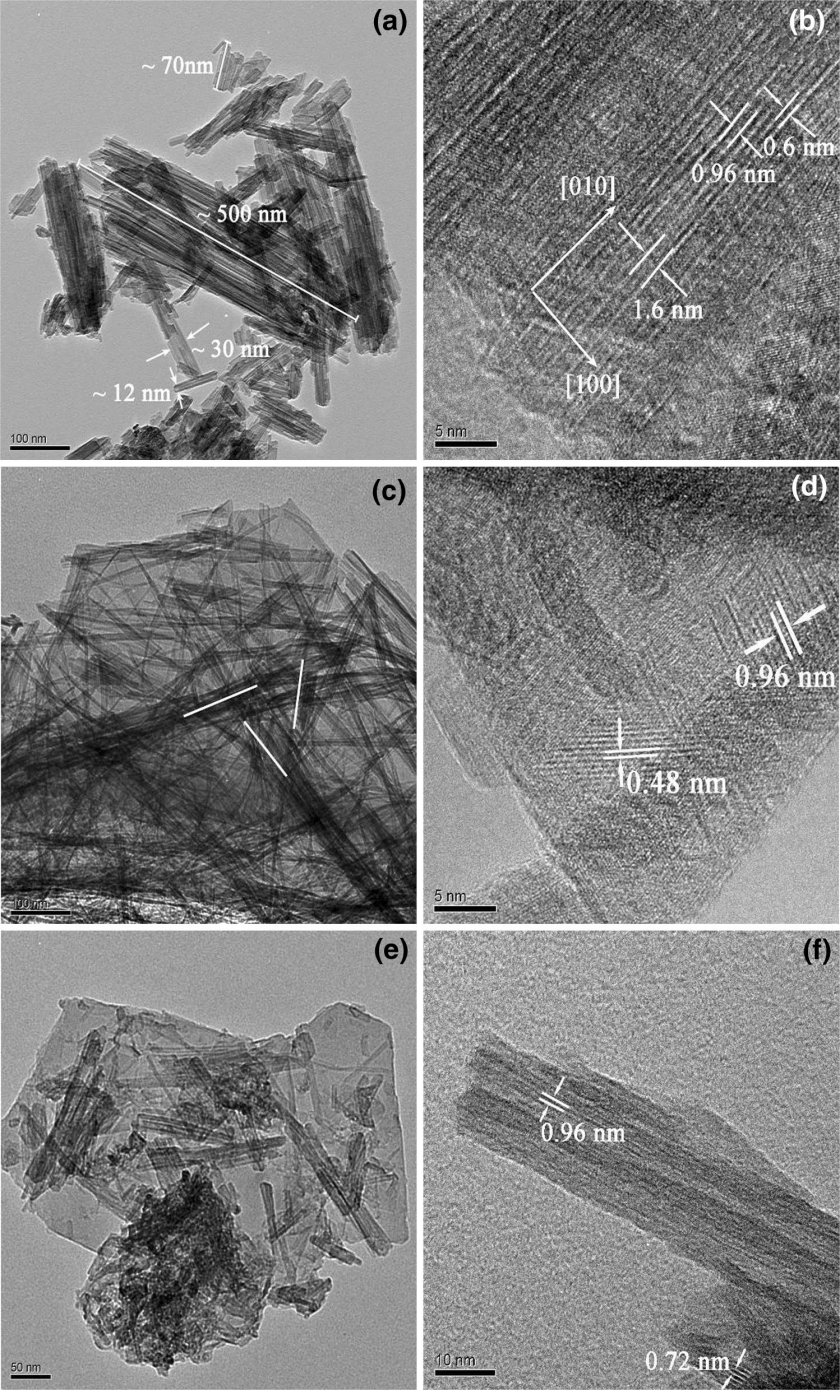
HR-TEM images of “*c*-disordered” Na-H-birnessites with different Mn(VII)/Mn(II) ratios after Mg-exchange and reflux treatment for 72 h. **a**, **b** 0.42-Na-H-Bir; **c**, **d** 0.52-Na-H-Bir; **e**, **f** 0.67-Na-H-Bir.

After 72 h reflux treatment of 0.42-Na-H-Bus, the formed todorokite consists of well-crystallized laths aggregating laterally across the (100) direction, measuring ~12–30 nm in width and ~70–500 nm in length along the (010) direction (Figure 5a). The main lattice fringe spacing along the (100) direction, i.e. the tunnel width, is 0.96 nm (Figure 5b), equivalent to three MnO_6_ octahedral chain widths. In addition, spacings of 0.6 and 1.6 nm, corresponding to two and four MnO_6_ octahedral widths are also observed, which is consistent with the reported by Atkins et al. [15]. This phenomenon of tunnel-width inconsistencies are commonly observed in natural and synthesized todorokite samples [11, 30–33]. The morphology of 0.52-Na-H-Bus after reflux treatment for 72 h is dominated by elongated fibers (~5 μm) of crystalline todorokite (not shown). These fibers appear to be aggregated into a dense network of fibers within a plate-like matrix (Figure 5c). The overlapping fibers are aligned with each other at 120° (Figure 5c), which is a characteristic morphology of the trilling pattern of todorokite. The lattice fringe spacings are 0.96 and 0.48 nm (Figure 5d). For 0.67-Na-H-Bus after reflux treatment for 72 h, small fibrous needles can be seen to be intergrown within the platy matrix (Figure 5e). Lattice fringe spacings of 0.96 and 0.72 nm can be observed (Figure 5f), implying that this sample may contain two phases of birnessite and todorokite. However, its XRD pattern does not show discernible reflections of todorokite, and so the content of todorokite maybe too small to be detected by XRD.

FTIR spectroscopy can conclusively distinguish between layer-type birnessite and tunnel-type todorokite [15, 34], because the broad peak at ~760 cm^−1^ in FTIR of Mn oxides is typically assigned to an asymmetrical Mn–O stretching vibration, corresponding to corner-sharing MnO_6_ octahedra, which is absent in phyllomanganate Mn oxides [34]. Figure 3b shows FTIR spectra of “*c*-disordered” Na-H-birnessites after Mg-exchange and reflux treatment for 72 h. Besides the common bands of Mn oxides at 438, 517, and 558 cm^−1^, the characteristic peak of todorokite at ~762 cm^−1^ can be observed with decreasing intensities from 0.42-Na-H-Bus 72 h reflux to 0.52-Na-H-Bus 72 h reflux to 0.67-Na-H-Bus 72 h reflux (Figure 3b). This result suggests that these “*c*-disordered” Na-H-birnessites are converted into todorokite to different extents after reflux treatment, and low Mn AOS is favorable to the transformation. This conclusion agrees very well with the XRD and TEM analyses (Figures 4, 5).

## Discussion

### The effect of interlayer Na^+^ and K^+^ on the formation of tectomanganates

It is believed that a small amount of cations such as Ca^2+^, Mg^2+^, Ni^2+^, Li^+^, Na^+^, K^+^, NH_4_^+^, or H_3_O^+^ is required to stabilize the tunnels in the formation of tectomanganates [35–37]. The order of hydrated radii of interlayer ions is K^+^ (3.31 Å) < Na^+^ (3.58 Å) < Mg^2+^ (4.28 Å). After the “*c*-disordered” H^+^-birnessite samples had been exchanged with Mg^2+^ for 24 h, most of the interlayer Na^+^ was replaced by Mg^2+^ (Table 2). Only part of the interlayer K^+^ was replaced by Mg^2+^ in the 0.52-K-H-Bus sample, probably caused by stronger electrostatic interaction between K^+^ with smaller hydrated radius and negative MnO_6_ octahedral layers compared with Na^+^ and Mg^2+^. The transformation from 0.52-K-H-Bus to todorokite is slower than that from 0.52-Na-H-Bus, which can be attributed to the fact that the interlayer K^+^ of 0.52-K-H-Bus cannot be completely exchanged with Mg^2+^, and the remaining 12.6% of interlayer K^+^ inhibits transformation of buserite to todorokite.

Under reflux treatment at 100°C, 0.52-H-Bus is transformed into ramsdellite, while Na/K-H-buserites are transformed into todorokite. This significant difference may be ascribed to different contents of interlayer ions in their precursors. The levels of Mg^2+^ and K^+^ in the interlayer of 0.52-H-Bus are 0.110 and 0.012 in molar ratios of Mg/Mn and K/Mn, or 0.233 equivalent charge of cations per Mn atom (Table 2). These values are much lower than the levels in 0.52-Na-H-Bus (0.273 and 0.005, or 0.554) and 0.52-K-H-Bus (0.234 and 0.033, or 0.501). Given the close MnO_6_ layer structures of 0.52-Na-H-Bus, 0.52-K-H-Bus, and 0.52-H-Bus, it can be inferred that a substantial part of the negative layer charge of 0.52-H-Bus is also electrostatically balanced by H^+^, either structurally bound or free in interlayers, apart from Mg^2+^ and K^+^ ions in the interlayers. Because of the smaller hydrated radius of H^+^ (2.82 Å) compared to K^+^, Na^+^, and Mg^2+^, the interaction between H^+^ and birnessite layers is supposed to be the greatest, which can account for the lower Mg^2+^ content of 0.52-H-Bus in comparison with 0.52-K-H-Bus and 0.52-Na-H-Bus (Table 2). Ramsdellite has a 1 × 2 tunnel structure, which is constructed of double chains of octahedra linked with single octahedral units by sharing corner oxygen atoms to form a rectangular cross-section [9]. Waychunas [38] reported that the tunnels of ramsdellite are normally empty but trace amounts of water, Na, and Ca might be present in the tunnels. Todorokite has a 3 × 3 tunnel structure with corner-sharing triple chains of MnO_6_ octahedra that are saturated with different larger cations such as Mg^2+^, Ca^2+^, and Ni^2+^ [3]. The large tunnels (6.9 × 6.9 Å) make todorokite materials possess a high ion-exchange capacity [4]. Therefore, lower contents of interlayer metal ions can account for the transformation of “*c*-disordered” H^+^-birnessite to ramsdellite with a smaller tunnel size of 1 × 2 rather than todorokite.

### The role of Mn AOS in transformation of “*c*-disordered” H^+^-birnessite into todorokite

The results show that from 0.42-Na-H-Bus to 0.52-Na-H-Bus to 0.67-Na-H-Bus, increasing reflux treatment time is required for both todorokite appearance and complete formation (Figure 4). In other words, “*c*-disordered” H^+^-birnessites with small Mn AOS values are readily transformed into todorokite, which is consistent with triclinic birnessite transformation to todorokite [8]. When Mg^2+^ exchanged “*c*-disordered” H^+^-birnessite is refluxed at 100°C, the Mn(III) species may easily move from the layers of buserite to the interlayer, leaving more vacancies in the buserite framework. Subsequently more interlayer Mn(III) species can gradually bond through covalence via condensation and dehydration reactions with each other and structural rearrangement to construct the “walls” of the tunnels. At this time, if the content and type of interlayer cations are sufficient to stabilize the 1-nm interlayer spacing, todorokite with 1-nm tunnel size will gradually form. Otherwise if the interlayer cations possess small hydrated radii, such as Na^+^, K^+^, or are present in insufficient amounts, such as in the case of 0.52-H-Bus, the 1-nm interlayer spacing will collapse and the shorter tunnel “walls” may be built instead. This leads to formation of tectomanganates with small size tunnels, for instance, cryptomelane with 2 × 2 size [39], ramsdellite with 1 × 2 size, or even pyrolusite with 1 × 1 size [40].

In addition, a sufficient amount of large interlayer cations does not necessarily facilitate the formation of todorokite if the Mn AOS of the precursor birnessite is high enough or there is insufficient Mn(III) species. After Mg^2+^-exchange for 24 h, the Mg^2+^ content of Na-H-Bus increases with increasing Mn(VII)/Mn(II) ratio in the synthesis of “*c*-disordered” H-birnessite (Table 2). This is probably because the Na-H-birnessite with large Mn AOS contains more vacancy sites [41, 42], and so more negative layer charge will develop and more Mg^2+^ is needed to reach charge equilibrium. However, the extent and rate of transformation into todorokite sharply decreases with increasing Mg content from 0.42-Na-H-Bus to 0.52-Na-H-Bus to 0.67-Na-H-Bus (Figure 4; Table 2).

It should be noted that “*c*-disordered” H^+^-birnessite is more difficult to convert to todorokite compared to triclinic birnessite with similar Mn AOS value under the same reflux conditions. For instance, triclinic birnessite with Mn AOS of 3.58 (0.33-Na-bir) can be almost completely converted into todorokite within 24 h [8], while it will take 48 h for complete conversion of 0.42-Na-H-Bir with Mn AOS of 3.55. One explanation for this could be that greater structural adjustment may be required for “*c*-disordered” H^+^-birnessite, a type of hexagonal birnessite, to be converted into todorokite relative to triclinic birnessite. Our other experiments showed that hexagonal birnessites, either δ-MnO_2_ or acid birnessite, with high Mn AOS of 3.85 are barely converted into todorokite after Mg^2+^ exchange and reflux treatment (data not shown). After reacting with low-concentration aqueous Mn(II), these hexagonal birnessites can gradually transform into birnessites with a orthogonal layer symmetry, and the produced birnessites can be completely converted into todorokite after 24 h of reflux treatment. This result confirms that triclinic birnessite with orthogonal layer is more likely than hexagonal birnessite to be converted into todorokite. Thus, triclinic birnessite had been exclusively used to prepare todorokite in the laboratory in the previous literature until recently Atkins et al. [15] reported successful synthesis of todorokite using “*c*-disordered” H^+^-birnessite as the precursor.

Ideal hexagonal birnessite hardly contains Mn(III), i.e. it has high Mn AOS but a large number of vacancy sites (16.7%), contrarily ideal triclinic birnessite barely possesses vacancy sites but does have a large amount of Mn(III) (33.3%) [17, 23, 43]. In this study, with decreasing Mn AOS the layer structure of “*c*-disordered” birnessite may adjust gradually towards that of triclinic birnessite, in other words, “*c*-disordered” birnessite with low Mn AOS may modify its structure, at least partially, towards triclinic birnessite through layer symmetry adjustment. These will definitely facilitate their transformation to todorokite. Further study is needed to explore the pathway of todorokite formation and whether triclinic birnessite is the necessary intermediate during the transformation of hexagonal birnessite into todorokite.

Therefore, Mn(III) together with content and type of interlayer metal ions plays a crucial role in the transformation of “*c*-disordered” H^+^-birnessite with hexagonal symmetry to todorokite, which is also the case for the triclinic birnessite transformation [8]. These results provide a further explanation for the common occurrence of todorokite in the hydrothermal ocean environment [32, 44], where is usually enriched in large metal ions such as Mg, Ca, Ni, Co and etc., and plenty of Mn(II) released from hydrothermal activity. This Mn(II) can easily cause Mn(III) formation in birnessite, either with orthogonal or hexagonal layer symmetry.

## Conclusions

The transformation of a layered birnessite to a tunnel-structure todorokite is a complex process. From the above results it is evident that the phase transformation of layer-structure “*c*-disordered” H^+^-birnessite to tunnel-structure todorokite is greatly affected by the amount of Mn(III) and the type and content of interlayer cations in the birnessite. Under reflux conditions, the degree of transformation of “*c*-disordered” H^+^-birnessite to todorokite decreases significantly as the Mn AOS values of Na-H-birnessite increase from 3.58 to 3.74. The transformation from K-H-birnessite to todorokite is slower than that from Na-H-birnessite, because the interlayer K^+^ of birnessite cannot be completely exchanged with Mg^2+^, and so interlayer K^+^ inhibits transformation of birnessite to todorokite. “*c*-disordered” H^+^-birnessite contained lower levels of Na/K is transformed into ramsdellite rather than todorokite.
